# The molecular mechanism of cell cycle arrest in the Bursa of Fabricius in chick exposed to Aflatoxin B_**1**_

**DOI:** 10.1038/s41598-018-20164-z

**Published:** 2018-01-29

**Authors:** Ping Hu, Zhicai Zuo, Hang Li, Fengyuan Wang, Xi Peng, Jing Fang, Hengmin Cui, Caixia Gao, Hetao Song, Yi Zhou, Zhengli Chen

**Affiliations:** 1 Key Laboratory of Animal Diseases and Environmental Hazards of Sichuan Province, College of Veterinary Medicine, Sichuan Agricultural University, Chengdu, Sichuan 611130 PR China; 2 College of Veterinary Medicine, Sichuan Agricultural University, Chengdu, Sichuan 611130 PR China; 3 College of Life Sciences, China West Normal University, Nanchong, Sichuan 637002 PR China; 4 Life science department, Sichuan Agricultural University, Yaan, Sichuan 625014 PR China

## Abstract

Aflatoxin B_1_ shows potent hepatotoxic, carcinogenic, genotoxic, immunotoxic potential in humans and many species of animals. The aim of this study was to clarify the underlying mechanism of G_0_G_1_ phase and G_2_M phase arrest of cell cycle in the bursa of Fabricius in broilers exposed to dietary AFB_1_. 144 one-day-old healthy Cobb broilers were randomly divided into two groups and fed on control diet and 0.6 mg·Kg^−1^ AFB_1_ diet for 3 weeks. Histological observation showed that AFB_1_ induced the increase of nuclear debris and vacuoles in lymphoid follicle of BF. Results of flow cytometry studies showed that bursal cells arrested in G_2_M phase at 7 days of age and blocked in G_0_G_1_ phase at 14 and 21 days of age following exposure to AFB_1_. The qRT-PCR analysis indicated that cell cycle arrested in G_2_M phase via ATM-Chk2-cdc25-cyclin B/cdc2 pathway, and blocked in G_0_G_1_ phase through ATM-Chk2-cdc25-cyclin D/CDK6 pathway and ATM-Chk2-p21-cyclin D/CDK6 route. In a word, our results provided new insights that AFB_1_ diet induced G_2_M and G_0_G_1_ phase blockage of BF cells in different periods, and different pathways were activated in different arrested cell cycle phase.

## Introduction

Aflatoxin B_1_ (AFB_1_), secondary metabolites generated by the fungi *Aspergillus flavus* and *Aspergillus parasiticus*, usually can contaminate agricultural products and threaten food safety^1^. AFB_1_ also presents potent hepatotoxic, carcinogenic, genotoxic, immunotoxic potential^2,3^ and other adverse effects in many species of animals, including rodents, fish, humans and non-human primates^4^. Immunosuppression is a major effect of AFB_1_, which is characterized by injuries of mucosal immunity, cellular immunity and humoral immunity. These injuries include alteration of organ morphology and immune organ weights, reduction of T or B lymphocytes number, inhibition of lymphocyte activity^5,6^, decrease of antibody production^7^, changes of T lymphocyte subsets of peripheral blood^8,9^, and increased sensitivity of poultry to bacterial^10^, viral and protozoan diseases^11^.

In order to clarify the mechanisms of AFB_1_-induced toxicity, many researches have been focused on the mechanism of cell cycle arrest in different cells. Previous studies have shown that AFB_1_ could induce G_2_M phase arrest in broiler’s jejunum *in vivo*^12,13^; S-phase accumulation in human bronchial epithelial cell *in vitro*^14^; G_0_G_1_ phase blockage in hepatocytes of rat *in vivo*^15^. Moreover, AFB_1_ can arrest immune cells particularly lymphocytes growth at G_2_M phase *in vivo*^16^. Further researches indicated that AFB_1_ can affect cell cycle through different signaling pathways. For example, AFB_1_-induced S-phase arrest might be mediated via inhibiting Wnt/β-catenin signaling route in HepG2 cells *in vitro*^17^, however, it may be triggered by activating ATM/ATR, Chk2, and p53 signaling pathways in human bronchial epithelial cells^14^. Yin *et al*.’s study indicated that AFB_1_ induced G_2_M phase arrest via ATM-Chk2-cdc25-cyclin B/cdc2 route in jejunum of broilers *in vivo*^13^. In rat models^15^, upregulation of miR-34a-5p led to cell cycle arrest at G_0_G_1_ phases via inhibiting cell cycle-related genes (CCND1, CCNE2 and MET) after exposed to AFB_1_. Although previous studies have shown that aflatoxin-contaminated corn induced G_2_M phase blockage in bursal cells^18^ and splenocytes^8^ of chickens, and G_0_G_1_ phase arrest in thymocytes^18^ of broilers, the molecular mechanisms and signaling pathways of cell cycle arrest of bursal cells have not been mentioned.

Bursa of Fabricius (BF), peculiar central lymphoid organ of birds, has major roles in establishment and maintenance of B cell compartment and humoral immunity^19^. In the present investigation, a broiler model was used to clarify the signaling pathway related molecular mechanisms involved in the cell cycle arrest of BF cells after dietary AFB_1_ treatement. We analyzed the histological lesions of BF, cell cycle phase distribution of bursal cells, mRNA expression levels of regulatory molecules involved in G_0_G_1_, S and G_2_M transitions, and the protein expression level of proliferating cell nuclear antigen (PCNA).

## Results

### Histopathological lesions of BF

**Figure 1:**
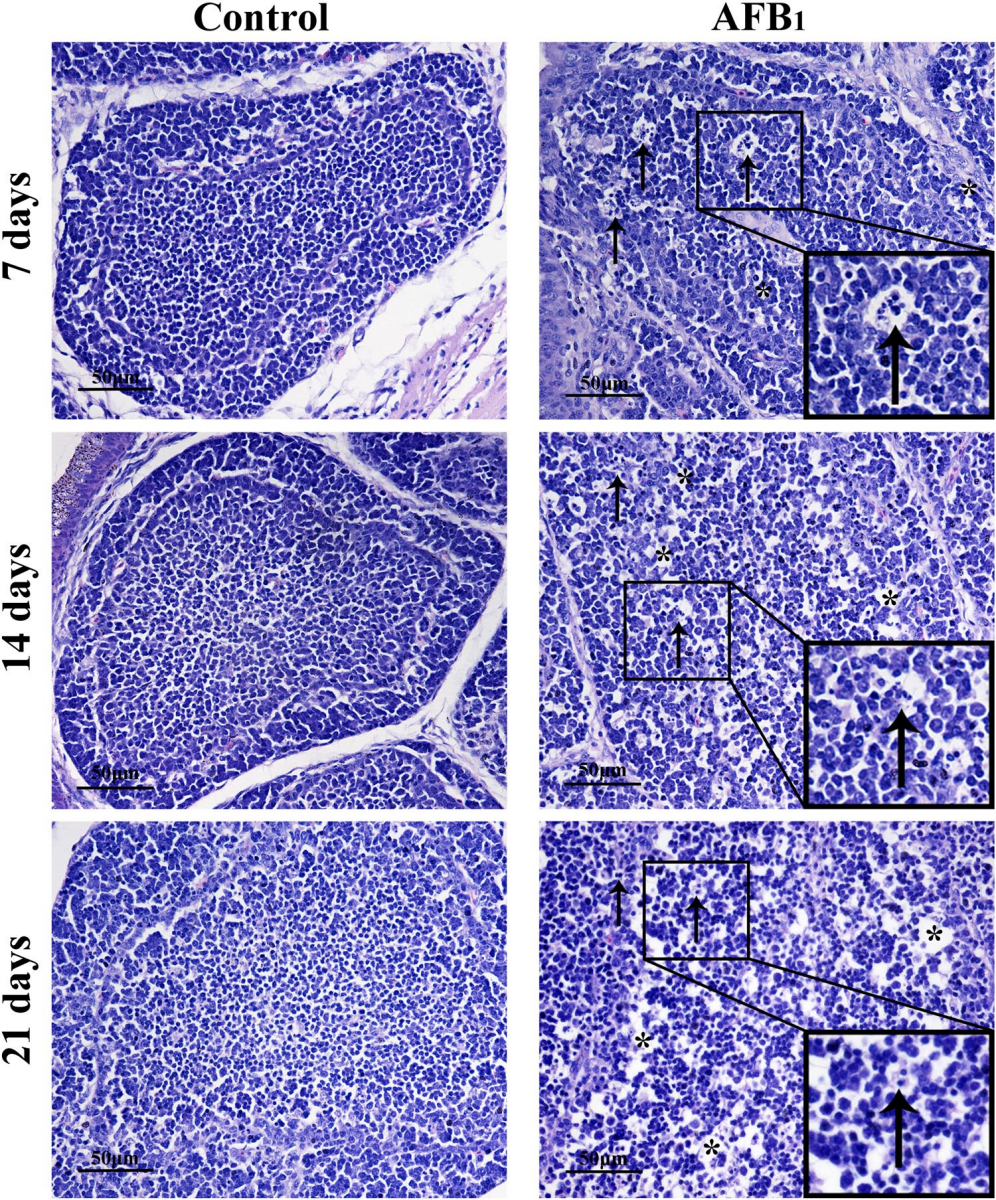
The impact of AFB_1_ exposure on BF’s histopathological (nuclear debris and vacuoles). Histological assessment of H&E-stained BF tissues of broilers at 7, 14 and 21 days of age in the control group and AFB_1_ group. ^↑^Marks nuclear debris in BF, *increased number of vacuoles.

Histopathologically, there were much more nuclear debris and vacuoles in cortical and medullary areas of bursal follicles in the AFB_1_ group when compared with those of the control group at 7 and 14 days of age. At 21 days of age, the population of lymphocytes was decreased, and tissue cells in the medulla loosely arranged, but there were not so much nuclear debris as those at 7 days of age (see Fig. 1).

### Cell cycle phase distribution of bursal cells

**Figure 2:**
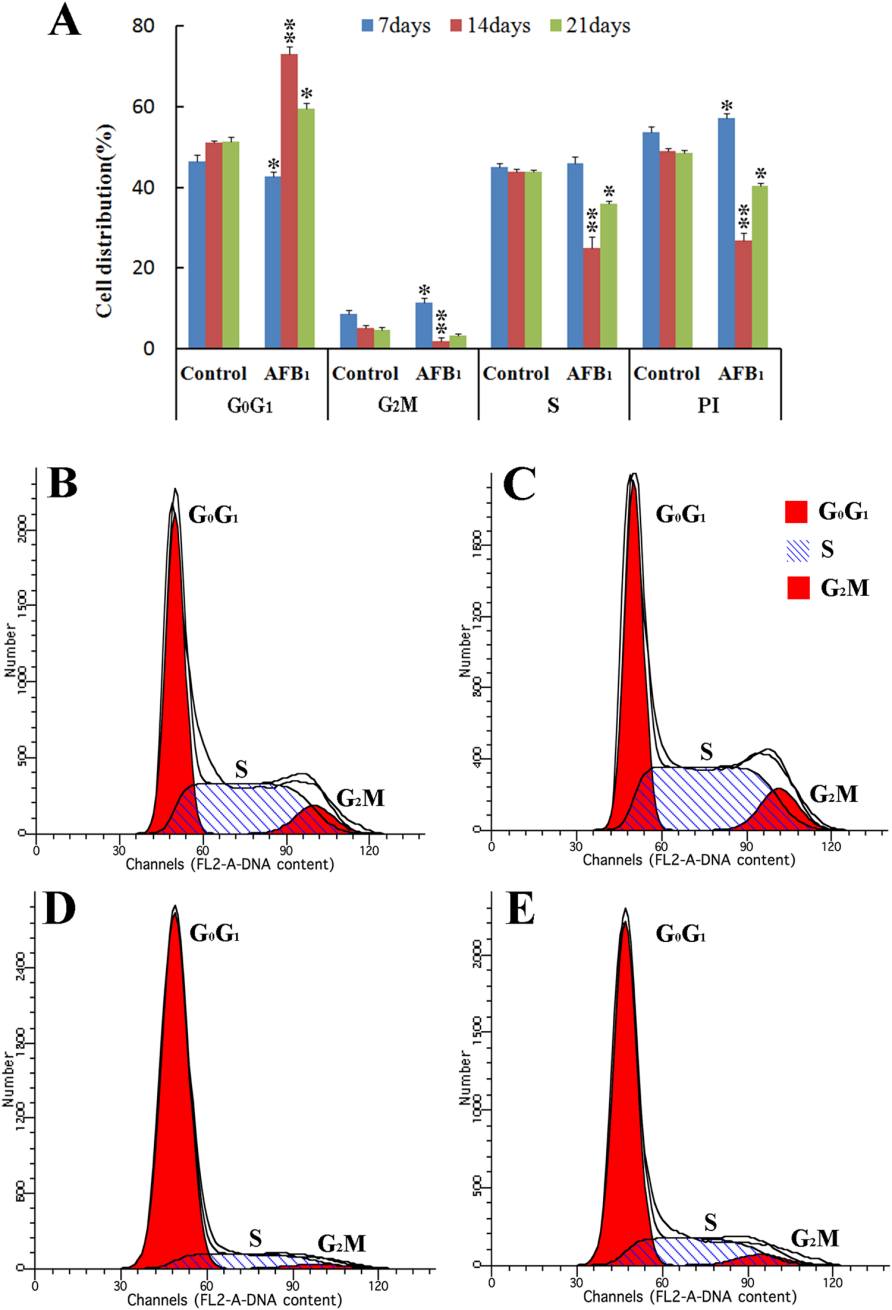
Effect of AFB_1_ on cell cycle phase distribution of BF in chickens. Bar graph (**A**) shows the change of percentages of G_0_G_1_, S and G_2_M phase distribution of BF cells. Data are presented as means ± standard deviation (n = 6). Letters *mean *p* < 0.05 and **mean *p* < 0.01 between the AFB_1_ group and control group, respectively. Histograms by flow cytometry show that cell cycle arrest in G_2_M phase at 7 days of age (**C**) and blockage in G_0_G_1_ phase at 14 and 21 days of age (**D** and **E**) in the AFB_1_ group when compared with those of the control group (**B**).

In the AFB_1_ group, the percentage of cells in G_0_G_1_ phase was lower than that of the control group at 7 days of age (*p* < 0.05), but was higher at 14 and 21 days of age (*p* < 0.05 or 0.01). The percentage of bursal cells in G_2_M phase was increased when compared with that of the control group at 7 days of age (*p* < 0.05), but was significantly decreased at 14 days of age (*p* < 0.01). In comparison to the control group, the percentage of bursal cells in S phase were decreased at 14 and 21 days of age (*p* < 0.05 or 0.01), but the change was not obvious at 7 days of age (*p* > 0.05). There were decreased tendency on PI values between the control group and AFB_1_ group at 14 and 21 days of age (*p* < 0.05 or 0.01), but the PI value was increased at 7 days of age (*p* > 0.05). These results showed that G_2_M phase was arrested at 7 days of age and G_0_G_1_ phase was blocked at 14 and 21 days of age in chicken’s BF. Histograms of cell cycle distribution by flow cytometer were shown in Fig. 2.

### Relative expressions of cell cycle-related genes

At 7 days of age, compared with the control group, the mRNA level of cdc2 was obviously decreased (*p* < 0.01), however, there were not significantly changes on the mRNA expressions of cyclin D_1_, cyclin E_1_, cyclin B_3_, CDK6 and CDK2 in the AFB_1_ group (*p* > 0.05). At 14 days of age, the mRNA contents of cyclin D_1_ and cyclin E_1_ were increased (*p* < 0.05), nevertheless, the expressions of CDK6, CDK2 and cdc2 were obviously decreased when compared with those of the control group (*p* < 0.01). In the AFB_1_ group, the mRNA expressions of CDK6 and CDK2 were obviously decreased when compared with those of the control group at 21 days of age (*p* < 0.01), but there were no significant changes for cyclin D_1_, cyclin E_1_, cyclin B_3_ and cdc2 (*p* > 0.05).

**Figure 3:**
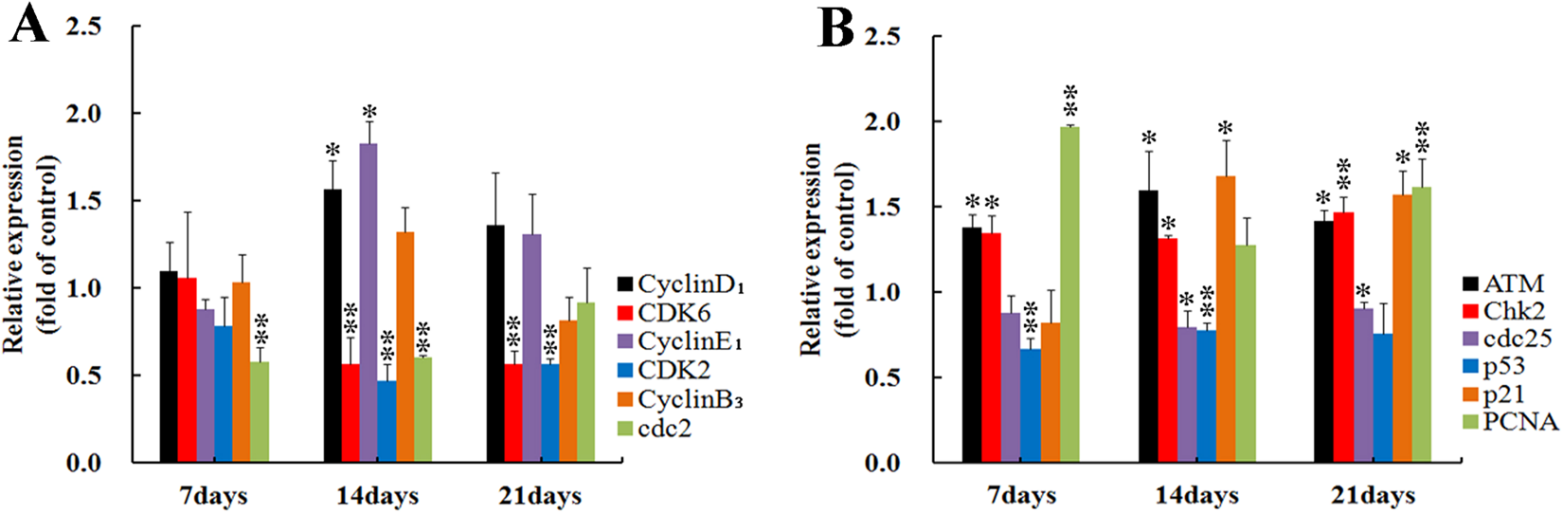
The relative expressions of mRNA in bursal cells from the broilers in the AFB_1_ group. Bar graph (**A**). The mRNA expressions of cyclin D_1_, CDK6, cyclin E_1_, CDK2, cyclin B_3_ and cdc2 in the bursal cells of the AFB_1_-fed broilers are expressed as fold change relative to the control-fed broilers. Bar graph (**B**). The mRNA levels of ATM, Chk2, cdc25, p53, p21 and PCNA in the bursal cells of the AFB_1_-fed broilers are expressed as fold change relative to the control-fed broilers. All data are expressed as means ± standard deviation. *p < 0.05, **p < 0.01 *vs* control, *n* = 6 for each group.

Compared with those of the control group, the mRNA levels of PCNA were significantly increased at 7 and 21 days of age (*p* < 0.01), the mRNA levels of p53 were obviously decreased at 7 and 14 days of age (*p* < 0.01). However, the expressions of p21 were increased at 14 and 21 days of age (*p* < 0.05), but there was no obvious change at 7 days of age. Furthermore, the mRNA expressions of ATM and Chk2 were higher at 7, 14 and 21 days of age than those in the control group (p < 0.05 or 0.01), and the mRNA levels of cdc25 were lower at 14 and 21 days of age (*p* > 0.05). The results were shown in Fig. 3 and Supplementary Fig. S1. The representatives of amplification curves of aforementioned 12 genes at 14 days of age were displayed in Supplementary Fig. S2.

### PCNA Expression

**Figure 4:**
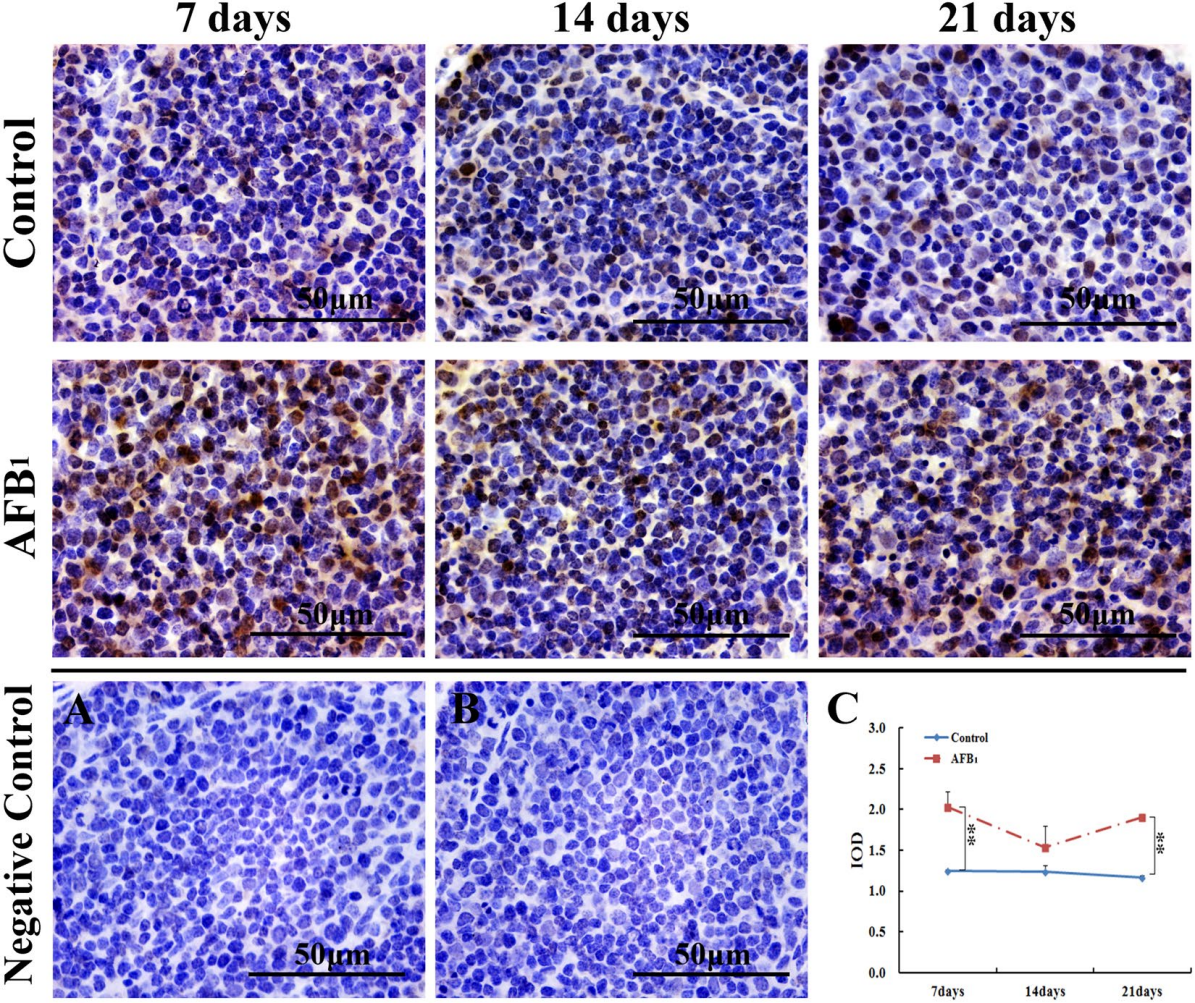
Protein expression of PCNA in bursal cells from the broilers in the control group and AFB_1_ group. Image (**A** and **B**) are representatives of the negative control staining in the control group and AFB_1_ group at 7 days of age, respectively. Line chart (**C**). shows the change of integrated optical density (IOD) of PCNA-positive cells by immunohistochemical method. All data are expressed as means ± standard deviation. **p < 0.01 *vs* control, *n* = 6 for each group.

As shown in Fig. 4, the nuclei of PCNA-positive cells were stained with brown color by immunohistochemical method. Compared with the control group, the numbers of PCNA-positive cells were increased in the AFB_1_ group. According to Fig. 4C, the IOD of PCNA-positive cells were found to be significantly increased in the AFB_1_ group at 7 and 21 days of age.

## Discussion

BF is primary central lymphoid organ of chicken^20^, which is related to diversification of B cells^19^. Histopathologically, consistent with our earlier studies^8,18,21^, obvious lymphocyte depletion, more vacuoles and debris were observed in the BF in the AFB_1_ group. It has been reported that chickens with poorly developed bursal follicles are expected to be very sensitive to bacterial^22^ and viral^23^ diseases, because lymphoid follicles of BF have a crucial roles in humoral immune reactions^24^. So these histological lesions could finally impaire the humoral immune function in chickens after exposed to AFB_1_.

Cell cycle is divided into different phases, including G_0_, G_1_, S, G_2_ and M phases. By FCM method, different phases of cell cycle are normally determined based on DNA content^25^. Many studies showed that AFB_1_ induce cell cycle arrest at different phases depending on the cell types, such as the accumulation of G_2_M phase cell in thymocytes^26^ and broiler’s jejunal epithelia^13^, the increase of S-phase cell population in human bronchial epithelial cells^14^, and the arrest at G_0_G_1_ phases in lymphocytes^16^ and liver cells^27^. Our previous study have shown that the percentage of bursal cells in the G_2_M phase was increased after treated with AFB-contaminated corn^18^. However, the present study showed that consumption of 0.6 mg·kg^−1^ AFB_1_ diet induced the arrest at G_2_M phase in bursal cells of broilers at 7 days of age and the blockage at G_0_G_1_ phases at 14 and 21 days of age. The results showed an interesting finding that the characteristics of cell cycle arrest could be changeable in the same kinds of cells with the increase of the AFB_1_ exposure time. Similar results have also been reported in splenocytes and thymocytes^8,18^. Why were the bursal cells arrested in different cell cycle phase at different period after exposed to AFB_1_? The transcription and translation of different cyclin genes may be related to the mechanisms of different cell cycle phase arrest. To further investigate the possible mechanism, the expressions of some cyclin genes were detected with qRT-PCR method.

Traditionally, as we know that the ATM-Chk2-cdc25 route and ATM-p21-p53-dependent pathway are the two classical pathways in cell cycle progression. Briefly, Ataxia telangiectasia mutated (ATM) is the primary kinase activated by DNA double-strand breaks (DSBs), which mediates the downstream signal cascade leading to cell cycle slowdown, DNA repair, and chromatin remodeling^28^. At the same time, in the process of DNA-damage, Chk2 is phosphorylated by phospho-ATM^29^, and then the phospho-Chk2 inhibits the activity of cdc25^30,31^. On the other hand, cyclin/CDK complexes are able to activate cdc25. More importantly, AFB_1_ can generally be metabolized by cytochrome P450 (CYP450) enzymes to generate active AFB_1_-8,9-epoxide (AFBO)^32^, which can react with DNA to form AFB_1_-FAPY adducts after forming AFB_1_-N7-Gua adducts^4^. The accumulation of AFB_1_-DNA adducts often induce DNA damage^14^. In the present study, the increase of ATM and Chk2 mRNA expressions, and the decrease of cdc25 mRNA level were observed at 7 days of age. Notably, the cyclin B_3_/cdc2 complexes were maintained at a relatively low level when compared with those of the control group. These results suggested that AFB_1_ activated ATM-Chk2-cdc25 pathway to inhibit cyclin B_3_/cdc2 expression in G_2_M phase. Similar results from other researchers were reported. Yin. *et al*.^13^ and Yang *et al*.^14^ showed that AFB_1_ induced accumulation of ATM and Chk2 in broiler’s jejunal epithelia and human bronchial epithelia *in vivo* and *vitro*, respectively.

p53, a tumor suppressor gene, has been shown to mediate cell cycle arrest, apoptosis and senescence in response to DNA damage^33,34^. p21 gene expression is generally under the transcriptional control of p53 protein^35^. However, the accumulated evidence indicates that p21-mediated cell cycle arrest can be either p53-dependent or p53-independent pathway^36–38^. Recent studies suggested that other unconventional cell cycle pathways are also involved in cell cycle progression. Namely, The full activation of Chk2 kinase can induce Chk2-dependent accumulation of p21^39^, and then induce cell cycle arrest at G_1_ phase through activating ATM-Chk2-p21 pathway in HaCaT cells *in vitro*^40^. In the current study, we found that the mRNA expressions of ATM, Chk2 and p21 were increased, and the expressions of p53 and cyclin D_1_/CDK6 complexes were decreased in the AFB_1_ group at 14 and 21 days of age. Interestingly, the expressions of cdc25 were also decreased when compared with that of the control group. These results indicated that AFB_1_ induce cell cycle arrest in G_0_G_1_ phase *via* two different routes at 14 and 21 days of age, namely ATM-Chk2-cdc25 pathway and ATM-Chk2-p21-p53-independent route.

p21 is a cyclin-dependent kinase inhibitor (CKI), which possesses the highest binding affinity among all PCNA-interacting partners^41^. Protracted p21 expression abrogated the property of PCNA to up-regulate CDKs and suppressed the PCNA mono-ubiquitination. Our results showed that AFB_1_ induced the increase of PCNA proteins and mRNA expressions. Because PCNA needs to be ubiquitinated for performing its biological function, these up-regulations of PCNA could not be certainly related to its promoted functions. On the other hand, p21 induces cell cycle arrest at G_1_^42^ and G_2_M^43^ phases by inhibiting the activity of CDKs such as CDK6, CDK2 and cdc2^42,44^. In this study, we found that the expressions of cyclin B_3_/cdc2 and cyclin D_1_/CDK6 complexs were decreased in the AFB_1_ group during the experiment. Therefore, maintaining a relatively low amounts of active cyclin/CDK complexes appears to be a major factor to regulate cell cycle progression^45^.

**Figure 5:**
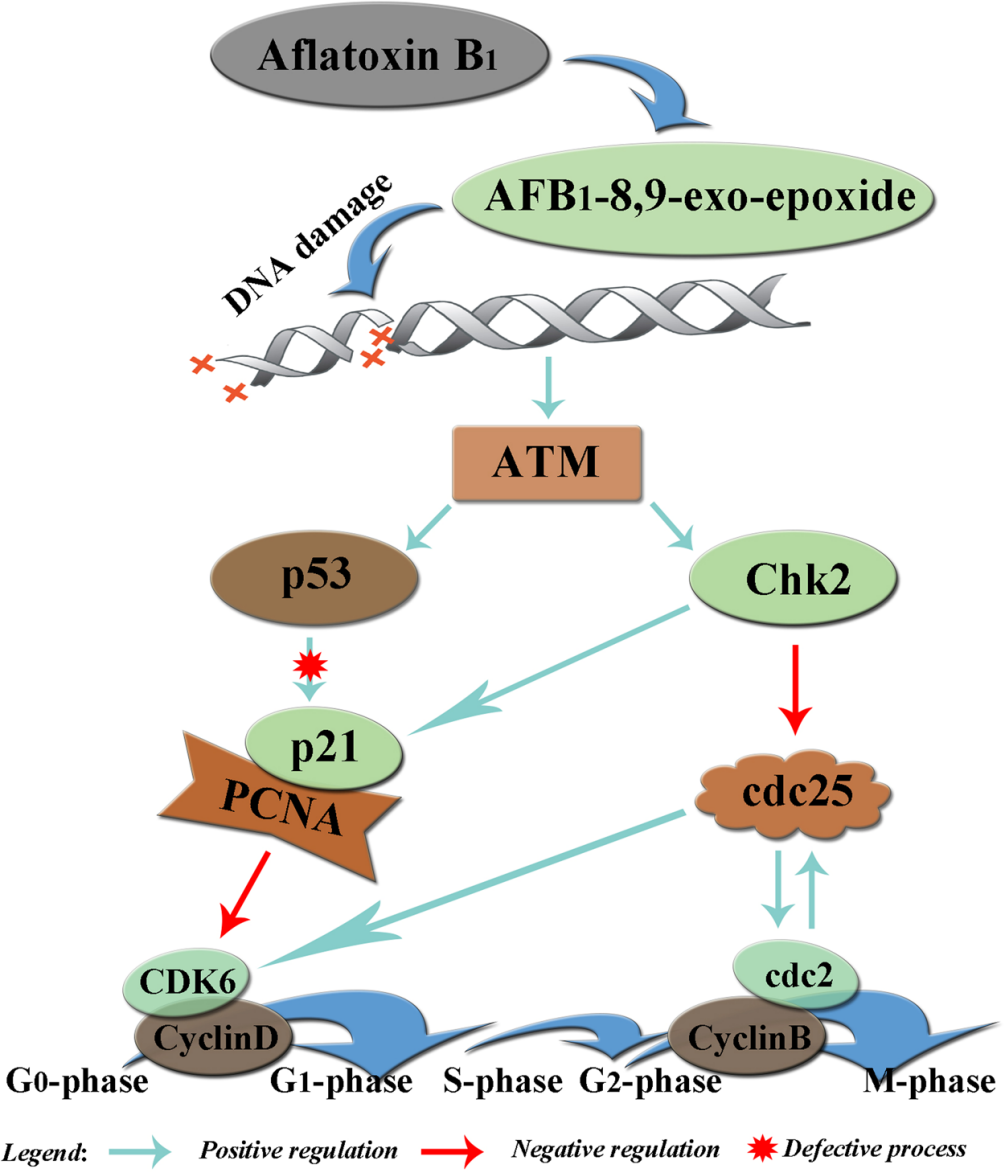
Schematic diagram of the proposed mechanisms of AFB_1_ induced cell cycle arrest in BF.

In summary, our results showed that 0.6 mg·kg^−1^ dietary AFB_1_ can induce histopathological lesions of bursa of Fabricius and cell cycle arrest in the bursal cells of broilers. Briefly, AFB_1_ induced G_2_M phase arrest *via* ATM-Chk2-cdc25-cyclin B/cdc2 pathway at 7 days of age, and G_0_G_1_ phase blockage through ATM-Chk2-cdc25-cyclin D/CDK6 pathway and ATM-Chk2-p21-cyclin D/CDK6 route at 14 and 21 days of age (see Fig. 5). Our results suggested that different mechanisms of G_0_G_1_ and G_2_M cell cycle might be involved in different stages of the development of BF, and could provide reference for deeper understanding of the mechanism of AFB_1_ induced immunosuppression for the same or similar studies in both human and other animals in the future.

## Materials and Methods

### Animals and diets

One hundred and forty-four one-day-old healthy Cobb broilers from Chia Tai Group (Wenjiang, Sichuan, China), weighed 40 ± 5 g, were randomly divided into two groups, namely control group (0 mg·kg^−1^ AFB_1_) and AFB_1_ group (0.6 mg·kg^−1^ AFB_1_). Each group consisted of three replications with 24 birds per replication. The control group received corn-soybean basal diet, which was formulated according to the National Research Council (NRC, 1994)^46^ and the Chinese Feeding Standard of Chicken (NY/T33-2004) recommendations, and the AFB_1_ group was fed with AFB_1_ diet. The AFB_1_ diet was made according to the method described by Kaoud *et al*.^47^. Briefly, 27 mg AFB_1_ (A6636, Sigma-Aldrich, USA) farinose solid was dissolved into 30 ml methanol completely, and then the mixture was mixed into 45 kg basal diet to formulate the AFB_1_ diet. The equivalent methanol was mixed into the basal diet to produce control diet. Afterward, the methanol of diets was evaporated at 98 °F (37 °C). The AFB_1_ concentrations were analyzed by HPLC with fluorescence detection (Waters Model 2475), and the AFB_1_ concentrations were determined as <0.01 mg·kg^−1^ for the control diet and 0.601 mg·kg^−1^ for the AFB_1_ diet. Broilers were housed in cages with electrically heated units and provided with water as well as aforementioned diet *ad libitum* for 21 days. Previous studies indicated that deficient or a complete lack of a functional glutathione-S-transferase (GST) with affinity toward AFBO appears to be a major reason that poultry are extremely susceptible to AFB_1_^48^. Additionally, 0.6 mg·kg^−1^ AFB_1_ in diet had obvious adverse effects on the immune organs of broilers, such as spleen^49^, thymus and BF^50^. Based on these information, experimental model (broiler) and toxin concentrations (0.6 mg·kg^−1^ AFB_1_) were chosen.

All study procedures followed the medical ethics according to national and international guidelines and has been approved by Sichuan Agricultural University Animal Care and Use Committee (Approval No: 2012-024).

### Histopathological examination

At 7, 14, and 21 days of age during the experiment, six broiler chickens were randomly selected from each group, and euthanized. The BF were immediately removed and fixed in 4% paraformaldehyde (PFA), embedded in paraffin, and sectioned at 5 μm. Some sections were prepared for immunohistochemistry, and some were stained with hematoxylin and eosin Y (H.E) for light microscopic observations. Typical histological changes were photographed with a digital camera (Nikon DS-Ril, Japan).

### Flow cytometry assay

At 7, 14 and 21 days of age during the experiment, six broilers in each group were sampled to determine the cell cycle phase distribution of BF cells by Flow Cytometry, with a similar method described by Chen *et al*.^51^. Single cell suspension of BF was harvested by dissecting each sample into small pieces and filtered through 300-mesh nylon gauze. Then, the cells were washed and suspended in phosphate buffered saline (PBS, pH 7.2~7.4) at a concentration of 1 × 10^6^ cells/mL. A total of 1 mL cell suspension was transferred into 5 mL culture tube and centrifuged at 800 × *g* for 5 min. Afterward, the cell suspension was permeabilized with 500 µL 0.25% Tritonx-100 at 4 °C for 20 min and centrifuged at 800 × *g* for 5 min. The supernatant was discarded, and then 1.5 µL of staining solution (Propidium iodide, BD Pharmingen, USA, 51-66211E) were added and incubated for 30 min at 4 °C in a dark room. Finally 400 µL of PBS was added and the cell cycle phase distribution was assayed by flow cytometry (BD FACSCalibur) within 45 minutes and analyzed by Mod Fit LT software for Mac V3.0 computer program.

$${\rm{Proliferating}}\,{\rm{index}}\,{\rm{value}}\,({\rm{PI}}\,{\rm{value}})=\frac{S+{G}_{2}M}{{G}_{0}{G}_{1}+S+{G}_{2}M}\times 100 \% $$

### qRT-PCR

BF from six birds in each group was removed and immediately stored in liquid nitrogen at 7, 14, and 21 days of age. Then, these BF samples were homogenized by crushing with a pestle and mortar until powdery. The powdered tissues were collected into eppendorf tubes and stored at −80 °C. As previously described^13^, total RNA was extracted from the powdery using TriPure Isolation Reagent (Cat No. 11667165001, Roche Applied Science, Germany) following the manufacturer’s instruction. Next, 1 µg of total RNA was used for reverse transcription using Transcriptor First Strand cDNA Synthesis Kit (Cat No. 04897030001, Roche Applied Science, Germany). For qRT-PCR reactions, cDNA was amplified using Bestar^®^ SybrGreen qPCR mastermix (Cat No. DBI-2043, DBI^®^ Bioscience, Germany) according to the manufacturer’s instruction for 40 cycles on a Bio-Rad C1000 Thermal Cycler (Step One Plus, Applied Biosystems, USA). Chicken β-actin was used as an reference gene^52^. Sequences for target genes (Table 1) were obtained from GenBank of NCBI and synthesized by Sangon Biotech (Shanghai, China). qRT-PCR data were analyzed by using the 2^−∆∆Ct^ calculation method^53^ and hierarchical cluster of gene expression data were analyzed by using HemI 1.0 software (Heatmap Illustrator, China).

**Table 1:** Primer sequence for proliferation genes.

Gene symbol	Accession number	Forward Primer (5′-3′)	Reverse Primer (5′-3′)	Product size
**ATM**	NM001162400.1	TTGCCACACTCTTTCCATGT	CCCACTGCATATTCCTCCAT	110bp
**cdc25**	NM001199572.1	AGCGAAGATGATGACGGATT	GCAGAGATGAAGAGCCAAAGA	163bp
**CDK2**	NM_001199857.1	TCCGTATCTTCCGCACGTTG	GCTTGTTGGGATCGTAGTGC	276bp
**CDK6**	NM_001007892.2	CCAGACCCGCACAACCTATT	TCTTGGCTGGATTGAACGCT	96bp
**cdc2**	NM205314.1	TCTGCTCTGTATTCCACTCCTG	ATTGTTGGGTGTCCCTAAAGC	144bp
**Chk2**	NM001080107	AGACCAAATCACTCGTGGAGAATAC	GATGCTCTAAGGCTTCCTCTATTGT	140bp
**cyclin D1**	NM_205381.1	GACTTTTGTGGCTCTGTGCG	CTGTTCTTGGCAGGCTCGTA	202bp
**cyclin E1**	NM_001031358.1	CGCCACCACAAAGCAGTAAG	TCACCGGCAGCATTTCCATA	137bp
**cyclin B3**	NM205239.2	ATCACCAACGCTCACAAGAAC	AGGCTCCACAGGAACATCTG	171bp
**p21**	AF513031.1	TCCCTGCCCTGTACTGTCTAA	GCGTGGGCTCTTCCTATACAT	123bp
**p53**	NM_205264.1	TGGAACCATTGCTGGAACCC	AGTTGCTGTGATCCTCAGGG	127bp
**PCNA**	AB053163.1	GATGTTCCTCTCGTTGTGGAG	CAGTGCAGTTAAGAGCCTTCC	104bp
**β-actin**	L08165	TGCTGTGTTCCCATCTATCG	TTGGTGACAATACCGTGTTCA	178bp

### Immunohistochemistry (IHC)

The immunohistochemistry technique used for PCNA was performed on 5 µm thickness sections according to the report by Yu *et al*.^54^. Briefly, the sections were deparaffinized and rehydrated. After washed three times with PBS (0.1 M, pH 7.2~7.4), the sections were treated with 3.0% hydrogen peroxide in PBS at room temperature for 10 min to quench the endogenous peroxidase activity. Following washed with PBS, the sections were exposed to normal goat sera for 30 min to block nonspecific antibody binding. In a humidified chamber, the sections were incubated the rabbit anti-PCNA polyclonal antibody (bs-0754R, Bioss, Beijing, China) for 20 h at 4 °C (working dilution: 1:100). After three successive washings in PBS, secondary antibody biotinylated goat anti-rabbit IgG and streptavidin-biotin complex (SA1020, Boster, Wuhan, China) were, in turn, applied onto sections for 1 h and 30 min at 37 °C, respectively. Slides were visualized with diaminobenzidine hydrochloride (AR1022, Boster, Wuhan, China) under the microscope and stopped by immersion in distilled water, as soon as brown staining was visible. Finally, the sections were lightly counterstained with hematoxylin and placed in absolute ethylalcohol and xylene for 3 min following coverslipped. Negative controls were performed in the same way, except that PBS was used as a substitute for the primary antibody.

The stained sections were photographed using a digital camera at ×1000 magnification. For each section, six randomly selected fields were used for analyzing integrated optical density (IOD) using Image Pro Plus 5.0 software (USA)^54^.

### Statistical Analysis

Statistical analyses were performed using SPSS 18.0 (SPSS Inc, Chicago, IL, USA). The experimental data were expressed as mean ± standard deviation ($\overline{X}$ ± SD) and independent sample test followed by post hoc *t* test was applied to determine the level of significance. Statistical significance was considered at *p* < 0.05 and markedly significant was considered at *p* < 0.01.

## Electronic supplementary material

Supplementary information
