# Supplementary material for: The molecular mechanism of cell cycle arrest in the Bursa of Fabricius in chick exposed to Aflatoxin B1
Source: Sci Rep. 2018 Jan 29;8:1770. doi: 10.1038/s41598-018-20164-z (PMC5789014; doi:10.1038/s41598-018-20164-z)
Supplement: Supplementary file 1 — Supplementary information [file 41598_2018_20164_MOESM1_ESM.pdf]

# The molecular mechanism of cell cycle arrest in the Bursa of Fabricius in chick exposed to Aflatoxin $B_1$

Ping Hu[1, 2, +], Zhicai Zuo[1, 2, +], Hang Li[1, 2, +], Fengyuan Wang[1, 2, +], Xi Peng[3, *], Jing Fang[1, 2, *], Hengmin Cui[1, 2], Caixia Gao[1, 2], Hetao Song[1, 2], Yi Zhou[4] & Zhengli Chen[2]

[1] Key Laboratory of Animal Diseases and Environmental Hazards of Sichuan Province, College of Veterinary Medicine, Sichuan Agricultural University, Chengdu, Sichuan 611130, PR China;

[2] College of Veterinary Medicine, Sichuan Agricultural University, Chengdu, Sichuan 611130, PR China;

[3] College of Life Sciences, China West Normal University, Nanchong, Sichuan 637002, PR China

[4] Life science department, Sichuan Agricultural University, Yaan, Sichuan 625014, PR China

[+] These authors contributed equally to this study.

[*] Correspondence and requests for materials should be addressed to: J. F. (email: fangjing4109@163.com) or X. P. (email: pengxi197313@163.com)

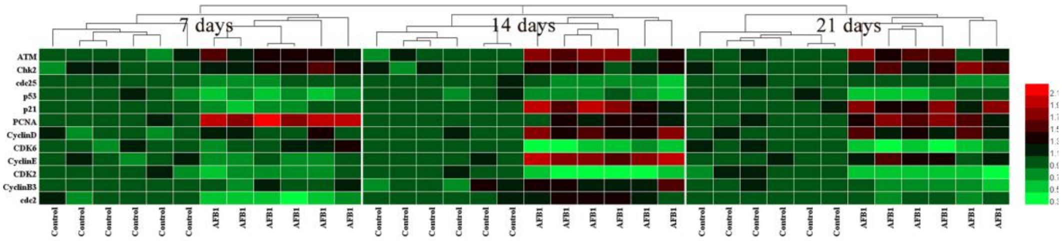


**Supplementary Figure S1.** Hierarchical cluster of gene expression data ($2^{-\Delta\Delta Ct}$) were analyzed by using HemI 1.0 software. Green boxes indicate lower expression than the control group expression and red boxes depict higher expression. (Green: low expression; Red: high expression; *n* = 6 for each group.)

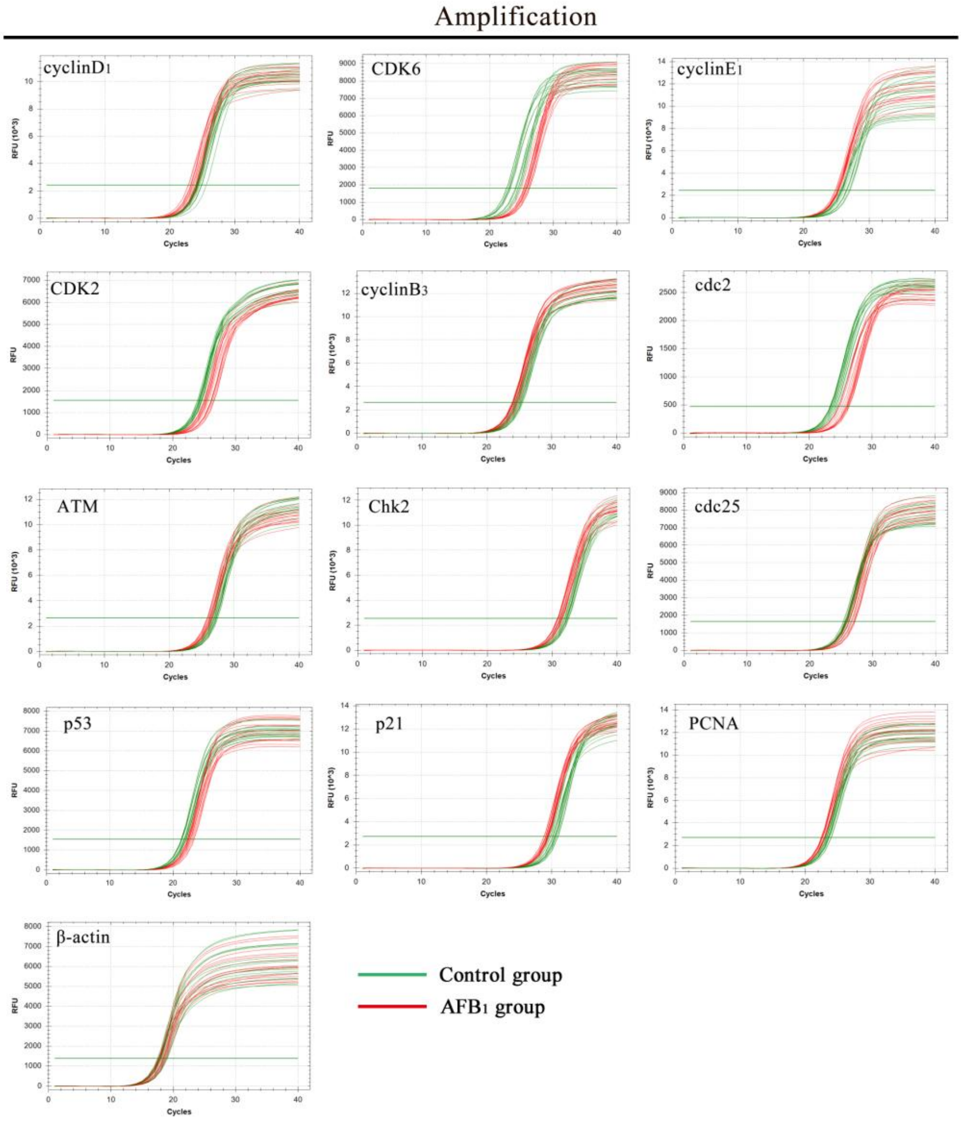


**Supplementary Figure S2.** Representatives of amplification curves of cell cycle-related genes (cyclin $D_1$, CDK6, cyclin $E_1$, CDK2, cyclin $B_3$ and cdc2, ATM, Chk2, cdc25, p53 and p21) and reference gene of chicken (β-actin) from the control group and the $AFB_1$ group at 14 days of age.
